# Women, intuition, and management—the Yin and Yang of nonconscious thought

**DOI:** 10.3389/fpsyg.2025.1572888

**Published:** 2025-09-12

**Authors:** Lois Isenman, Marta Sinclair

**Affiliations:** ^1^Brandeis University, Waltham, MA, United States; ^2^Griffith University, Brisbane, QLD, Australia

**Keywords:** women’s intuition, intuition and gender, gender and cognition, male vs. female intuition in management and leadership, encouraging women’s intuition in the workplace, intuition and interoception (bodily experience), wholeness and receptivity in women’s intuition

## Abstract

This article explores the claim that men’s and women’s intuition tend to differ in some way, and that women’s intuition can make a distinctive contribution to leadership and management. In response to the dynamic and unpredictable nature of the current business environment, intuition has received increased attention from the business community. Likewise, it has become an intense area of academic research, along with the unconscious knowledge and integration on which it largely depends. Although both women and men process information partially below awareness, there are likely some distinctions. Research shows that in many situations men tend to simplify information and focus primarily on its most salient aspects. In contrast, women tend to utilize information more completely, with greater sensitivity to the inter-relationships between different pieces, to context and to anomalies. We propose that there is an affinity between intuition and the way that women prefer to deal with information, and that much of their higher-level information processing and elaboration may occur below awareness. Recent work also indicates that women are more receptive to emotional and other body-based signals, which can help surface unconscious knowledge. Their greater focus on completeness and interconnections together with their emotional and bodily sensitivity tends to make them more open to additional information at both the unconscious and conscious level. Therefore, women’s intuition, although it can take time to germinate, has the potential to foster decision making, problem solving and human resources management that are more multifaceted and holistic. Male intuition, in contrast, seems to support expert behavior and efficiency. We suggest that taking advantage of women’s intuition in the workplace would require a twofold adjustment: women would need to gain confidence in their intuition and organizations would need to recognize its potential power.

## Introduction

In response to the dynamic and unpredictable nature of the business environment, intuition has received increased attention from the business community. During roughly the same period, it has become a focus of academic research, along with the unconscious intelligence on which it largely depends. Although this elusive mental mode is not restricted to women, it has often been associated with them. Indeed, for a number of historical and biological reasons explored in this article, women tend to be more comfortable with the idea of intuition than men (Tamblyn, 2022). Yet the possibility that the male and female approach to intuition tends to differ has received only a little academic attention.

In the recent past, interest in this question has been largely overshadowed by the important struggle for female equality (Cahill, 2017; Eagly, 1995). Although there is still a long way to go, it is a good time to consider whether women’s intuition might have something unique to contribute, not only in personal life, but also in the workplace. As we will show, there is various evidence, some old and some new, that suggests that this claim has merit. We appreciate that gender as well as gender-associated traits are not binary (Hyde et al., 2019) but bimodal, with large intra-gender variation indicating the potential influence of a variety of additional factors. Nonetheless, research on gender differences remains an important tool for understanding our full potential as humans (Cahill, 2017).

Investigating women’s intuition is particularly timely with respect to organizational issues. If the goal is to increase productivity by leveraging diversity, organizations need to utilize effectively the different attributes that each gender could contribute to the mix. The World Economic Forum more than a decade ago (World Economic Forum, 2010) proposed that female managers and leaders might introduce a style that is more long-term and includes non-monetary indicators to a larger extent. Similar conclusions were drawn from a recent meta-review of the executive gender literature (Mah et al., 2023). We would like to explore whether women in the workforce might indeed infuse a distinctive and important kind of intuition into decision making and leadership, which could work in a complementary fashion to the male kind with the aim to enhance the overall effectiveness of organizations.

Instead of defining intuition as a uniform concept, we offer a process-oriented characterization that brings together many different forms it can take. Intuition depends largely on the ability of the unconscious mind and its intelligence–specifically the cognitive unconscious (Kihlstrom, 1987) and many aspects of the psychodynamic unconscious (Ellenberger, 1970)–to take in and process information to make sense of our worlds (Isenman, 2018). It includes a hierarchy of mental activities that extends from simple pattern recognition, which is contiguous with animal intelligence (Cappon, 1994; Sutherland, 1968) to expert knowledge and culminates in the highest reaches of the human pattern creation capacity (Fox-Keller, 1983; Holton, 1973). These cognitive abilities depend on each other, with each expanding the potential of the one beneath it, as we will explore in more detail. Our interest lies most heavily on the aspect of intuition that is perhaps most important to organizations, its generative and creative capacity. For a variety of reasons that we will examine throughout the article, this is a capacity in which women might excel.

The process that unites the various forms of intuition is the way the unconscious mind tends to combine information: it interweaves multiple strands simultaneously to form a meaningful pattern. In contrast to consciousness, which can manage only 7 ± 2 different pieces of information at a time (Miller, 1956), the unconscious mind has unlimited capacity. Its holistic way of putting information together, which is at the core of pattern recognition, is variously labeled *parallel distributed processing*, *parallel interactive processing*, or, as we will often call it, intuitive processing or intuitive integration. It is holistic because it simultaneously merges both bottom-up and top-down ways of putting information together. This means that, in contrast to other ways of integrating information, the parts *automatically* determine the whole at the same time as the whole *automatically* determines which parts are included (Isenman, 2018).

Both men and women depend on intuitive processing for much, if not most, of their lower-level cognition and perception; however, research has identified some significant differences in the strategies that tend to shape their higher-level cognition from below the level of awareness. A number of studies that examine gender-based responses have shown that men tend to simplify the given information, focusing primarily on the apparently most salient or most relevant pieces. In contrast, women tend to utilize the information more comprehensively (Darley and Smith, 1995; Meyers-Levy, 1986; Meyers-Levy and Maheswaran, 1991; Meyers-Levy and Sternthal, 1991; Showkat and Grimm, 2018; Tsichla et al., 2016). They take into account more of the details, focus more on the relationship between different pieces of information (Daltrozzo et al., 2007; Putrevu, 2001; Wirth et al., 2007) and are more sensitive to anomalies (Meyers-Levy and Maheswaran, 1991; Meyers-Levy and Sternthal, 1991) as well as context (Gilligan, 1982; Kahai et al., 2023; Paraskeva et al., 2012; Stoet, 2017). All of this would enhance a woman’s ability to perceive complex novel patterns, so important to higher-level intuition. The evidence also suggests that women are more attuned to internal emotional and body-based signals (Alfano et al., 2023; Longarzo et al., 2021) that can play a vital role in intuition (Damasio, 1994; Isenman, 2018, 2020; Sinclair, 2020).

In Part I of what follows, we examine in more detail intuitive processing, or the holistic way the unconscious mind tends to integrate information. We argue there is a special affinity between intuitive processing and how women tend to process information and propose that their reliance on intuitive processing for higher-level understanding of the world is greater than men’s. In Part II, we focus on some of the research suggesting that men and women tend to favor different cognitive strategies for higher-level cognitive information processing, and we highlight the potentially unconscious nature of the additional information processing prevalent in women. In Part III, we explore women’s greater receptivity to emotional as well as internal bodily, or interoceptive, information and its integration with cognitive information in a region of the brain called the insula, which likely serves as an intuitive channel. In Part IV, we discuss how the greater immersion of women in their subject matter adds several additional levels of holism to their intuitions. In Part V, we contemplate how women’s unique kind of intuition, if integrated properly, may contribute to work productivity and shape a new kind of leadership.

## Part I: How the unconscious mind puts information together and its relevance to women

The unconscious mind prefers an intuitive way of putting information together, which allows each piece to interact with all the other pieces at the same time (Bechtel and Abrahamsen, 1991; Betsch and Glöckner, 2010). These multiple interrelationships provide the *meaning*, which is often all that comes to awareness. In contrast to this holistic way of integrating information, the conscious mind often favors a linear or step-by-step approach using the rules of ordinary logic.

Intuitive processing, which is at the core of pattern recognition (Bechtel and Abrahamsen, 1991), is responsible for intuitive perception, and thus for much of the way we naturally perceive the world and divide it into categories (Rosch, 1978). For example, we know a cat is a cat, and not a small dog or a rabbit, without thinking about it. Although the conscious mind might come up with a characteristic or two distinctive to cats, the unconscious mind makes the discrimination before the conscious mind has a chance. It depends on the interrelationships between its different characteristics that come together below awareness to form a pattern. The notion of *cat-ness*, or perhaps the label *cat*, just appears in the conscious mind, without us necessarily knowing at a conscious level the characteristics or their pattern of interconnection responsible for the recognition. These natural patterns also readily generalize to similar objects or events (Rosch, 1978); thus, knowledge of cat-ness can easily generalize from house cats to leopards and jaguars.

Intuitive integration or processing echoes the most basic structure of the nervous system in which each neuron links directly to a number of other neurons (y Cajal, 1899). This interwoven way of connecting information is the generally hidden internal structuring principle of intuition (Betsch and Glöckner, 2010), which is essential to the holism associated with it. Multiple pieces of information weave together below awareness to create a pattern that captures a sense of the whole—a gestalt conveying the meaning (Kohler, 1970), which is generally the only thing that needs to appear in consciousness.

However, pattern recognition is often possible with only partial information, due to its holism and grounding in past knowledge (Contrell, 1990; Sinclair and Ashkanasy, 2005). For example, it allows us to recognize a familiar face even when it is partially occluded by dark glasses, or sports a new, very different haircut. Since the available information taken together conveys a sense of the whole, it allows the mind to predict the missing information based on past experience. Because we recognize the whole pattern, we can fill in the missing details (Contrell, 1990). In certain situations, this can reduce to one or a few pieces of salient information being much more important than any of the others (Isenman, 2013). This has been termed *thin-slicing* (Ambady and Rosenthal, 1992), a notion popularized by Malcolm Gladwell in his book Blink (2005), which resonates with the male strategy of simplifying information. However, as we explore later, this understanding of “a blink of the eye” as a metaphor for intuition (Isenman, 1997) is only one of its many potential meanings.

A large portion of our basic perception is grounded in simple environmental pattern recognition, which lies on the lower level of the intuition continuum. For the most part, we do not “think” the world we perceive into existence. It appears fully formed from our unconscious mind with little or no conscious effort. This kind of direct knowing is contiguous with animal intelligence (Cappon, 1994; Sutherland, 1968). As such, it has been honed by evolution to work well within its realm much of the time.

The same ability in humans can support an increasingly complex and nuanced perception of the world. The expansion of our intuitive perceptual capacity depends on recording more complex new patterns in the environment, as well as generalizing old ones (Dane and Pratt, 2007; Gore and Sadler-Smith, 2011; Klein, 1998; Simon, 1987; Sinclair, 2010). Pattern recognition can also account for expert intuition, the knowledge that comes with increasing experience within a specific domain (Klein, 1998). The expansion of our intuitive prowess continues into the creative realm with the formation, or creation, of generative intuitive mental patterns. These can range from abstract patterns, which sometimes influence hypotheses in science and other fields (Boden, 2004; Hadamard, 1973), to the deeply resonant intuitive metaphors and similes that often just appear in consciousness and shape poetic expression (Boden, 2004).

Thus, the same principle that guides simple intuitive pattern recognition of the environment, such as recognizing a never-before-seen house cat as a cat, also guides the pattern formation involved in higher-level intuition; multiple pieces of information come together in a parallel interwoven way to form a subtle pattern of interrelationship. The details of the pattern frequently remain hidden, since in many circumstances the only thing that needs to come to awareness is a sense of the whole.

Sometimes generating a meaningful whole requires giving less weight to some pieces of information and more to others (Bechtel and Abrahamsen, 1991). Part of being an expert is knowing, largely below awareness, what information is more important (Klein, 1998, 2003). Yet, when an old pattern does not work, the key to the solution sometimes rests with the intuitive mind creating a new pattern by reconfiguring the old one around some of the information previously excluded or de-emphasized (Groopman, 2007; Isenman, 2013).

### Women may prefer intuitive processing for higher-level understanding

We are all experts in our own spheres. Both women and men use intuitive perception, or processing, to understand their environment and guide their actions. This is often adequate to extract the necessary information, especially when swift action is required (Dane and Pratt, 2007; Klein, 1998). Yet, when facing the struggle to forge novel mental ground, or to understand what is seemingly hard to get one’s mind around—the activity we generally refer to as thinking—women may depend more than men on parallel distributed processing, or intuitive processing, to weave together all the information below awareness (Bao et al., 2022; Ingalhalikar et al., 2013). Men, in contrast may be somewhat more likely to favor heuristics—i.e., simplified information processing—which can allow them to reach a conclusion quickly, or, alternatively, use conscious step-by-step analysis (Bao et al., 2022; Sladek et al., 2010), employing the most salient information for their premise.

Women may favor intuitive processing for putting higher level-information together because, as we argue is true of their preferred cognitive strategy, it is grounded in holism. We propose that this kind of processing and the female strategy both depend on a comprehensive use of the information based largely on its inherent interrelationships. Guided by the individual’s motivational system and goals, the details in their interaction with each other come together to create a sense of the whole. This wholeness comes not from the outline or circumference of the idea, but from the complex interconnection of the parts, or details (Isenman, 2018). Even though this interweaving of information tends to occur below awareness, what appears in consciousness nonetheless carries with it the richness and the feeling of coherence of the underlying integration. By the same token, this kind of holistic integration can also help to highlight information that is anomalous, or otherwise does not belong (Isenman, 2018; Mangan, 2001). In the following sections, we will explore more of the reasons why women may favor this kind of information processing for their higher-level cognition. But first we will focus on the pros and cons of such a cognitive strategy.

### Considering both sides of high-level intuitive integration

Women’s proposed inclination to interweave complex conceptual information outside of awareness suggests that, more often than men, they may find themselves knowing something important but have only a cloudy feel of this knowledge, even as they sense its correctness and importance. As a woman attempts to bring her understanding into consciousness, only bits and pieces may appear, while much remains below awareness (Isenman, 2018). Even when her insight seems clear and compelling to her, she may not be able to articulate her understanding cogently to others because of its complexity. Moreover, if challenged, she may not be able to justify her knowledge because its underpinnings remain hidden to her conscious mind. All this can make it difficult for women to offer their ideas or to convince others of them, especially in professional situations such as the boardroom.

Fortunately, whatever handicap using intuitive, or parallel interactive, integration for complex understanding might impose on a woman would be more than compensated for by its creative and predictive potential. As we stressed above, because the integration occurs largely below awareness, it can incorporate many more different strands of information than conscious processing (Betsch and Glöckner, 2010; Dijksterhuis and Nordgren, 2006), along with the potentially complex interactions between these multiple strands. Consequently, it can lead a woman to different, more holistic conclusions than either heuristics or step-by-step logical processing.

The integration can also include information that is unavailable to consciousness, such as implicit knowledge (Reber, 1993), once-conscious knowledge that has been forgotten (Isenman, 2018), as well as subtle bodily understanding that often goes unnoticed. The unconscious mind also has important additional capacities. With its tendency to use interconnected or relational processing in a relatively unrestrained manner, it can combine information around the more remote associations that are so important to the generation of creative ideas (Mednick, 1962). Moreover, the process is often guided by subtle emotional and other body-based signals, which, as we discuss later, women may be more attentive to (Alfano et al., 2023; Booth-Butterfield and Booth-Butterfield, 1990; Dunn et al., 2010; Grabauskaitė et al., 2017; Sinclair, 2020).

The different aspects of a woman’s intelligence can work together below awareness, combining input from her mind, her body and her emotions to inform her intuitive understanding. The brain can function in a parallel distributed or holistic way at many different levels of processing. Just as neurons are able to connect to many other neurons simultaneously, regions of the brain responsible for the various aspects of mental activity can interact simultaneously (Damasio, 1994; Pessoa, 2023). Working together, influencing and modifying each other, these different facets of intelligence can result in a deeper cognitive integration, and thus, as we argue, a broader sphere of wholeness.

### Intuitive thought and the relational focus of the female self

Another explanation for women’s predilection to use intuitive processing is that conscious intellectual power and its major tool, reason, were at least historically discouraged in women. As a result, women’s higher conceptual activity was more likely to happen below awareness. But it goes even deeper than that: intuitive processing with its relational, or parallel distributed, connectivity appears to be a better fit with a woman’s sense of self. As we will explore, it is more consistent with the way women perceive and interact with the world (Chodorow, 1978; Gilligan, 1982; Jordan et al., 1991; Miller, 1976).

The self organizes our mental life. It includes aspects we are conscious of, parts of the unconscious mind, as well as the aspects of body function that participate in cognition. It acts as an overarching filter that determines what we attend to and how we integrate it. The self helps to shape *what* information we take in, and it also may help to shape *how* we take it in (Belenky et al., 1986; Kahai et al., 2023; Palmer, 1988; Paraskeva et al., 2012; Stoet, 2017).

The relational focus of a woman’s sense of self is forged by both nature and nurture. With respect to nature, much of the science around gender and brain difference at this point still appears contradictory: for example, compare Eliot et al. (2021), to DeCasien et al. (2022), or to Ryali et al. (2024). Yet it is incontrovertible that *most* women are biologically prepared to be caregivers. With respect to nurture, women in their traditional role functioned as relationship builders and sustainers as well as caregivers. This required heightened emotional and subtle sensory awareness (Lyneham et al., 2008; Sinclair, 2020). The historical role of men in many cultures, in contrast, was of a protector and warrior, where action preparedness, along with the capacity for bold action, was all important and emotional considerations were often experienced as weakness (Chodorow, 1978).

Even though women and men now are less constrained to their traditional spheres, a woman’s sense of self still tends to be more focused on interdependence and connections with others, whereas a man’s sense of self is more focused on autonomy and individual agency (Brody and Hall, 2008; Vial and Cowgill, 2022). Indeed, women tend to have a somewhat higher density of anatomical as well as functional connections *between the two hemispheres* of their brains (Ingalhalikar et al., 2013; Cook et al., 2023), which would allow more efficient and effective integration of emotional and body-based cues with cognitive information (Meyers-Levy and Zhu, 2010) and support social cognition and relationship building. Men, to the contrary, have a somewhat higher density of *within hemisphere* connections (Ingalhalikar et al., 2013; Cook et al., 2023), which would support action preparedness and autonomy.

These contrasting gender tendencies are sometimes referred to as the Big Two in the research on gender differences (Martin and Slepian, 2021). They have been characterized in a number of different ways: for example, agency vs. communality, competitiveness vs. nurturing, competence vs. warmth, self-interest vs. other’s interest, transactional vs. moral. These juxtapositions can refer to somewhat different aspects of functioning (Martin and Slepian, 2021). Although they are not necessarily directly linked to each other (Eagly, 1995; Grissom et al., 2024), together they reflect the biological and social forces that have traditionally shaped gender identity.

Women’s nurturing, or caring focus, as we intimate above, requires attunement to others that is to a large extent achieved through registering and responding to their emotions. In accordance with this, women tend to have higher emotional awareness than men (Booth-Butterfield and Booth-Butterfield, 1990; Sinclair, 2020). Emotion, an evolutionary adaptation to support species survival (Darwin and Prodger, 1998), is an important potential precursor of intuition (Cappon, 1994; Damasio, 1994; Sinclair and Ashkanasy, 2005). For women, intuition is frequently expressed through their aptitude and propensity to care for others (Benner and Tanner, 1987; Rand et al., 2016; Vial and Cowgill, 2022). They are often required to integrate the emotional as well as physical needs of multiple others with their own. Indeed, as Verma and Kind (2025) point out in their paper, testosterone levels, which are of course higher in men, act to inhibit attunement to others. Women’s proclivity toward emotional receptivity and attunement to a potential variety of intersecting influences is probably part of the reason that women might favor intuitive, or relational, processing and are more accepting of intuition than men. As managers and leaders, their caring focus also enables them to factor in important considerations that are less quantifiable (Stirling, 2011; Vial and Cowgill, 2022; World Economic Forum, 2010). These include the impact on human capital and sustainability, reflected in the long-term well-being of employees, of the organization, and of society as a whole (Offermann and Foley, 2020; Stirling, 2011; World Economic Forum, 2010).

## Part II: Cognitive differences in the way men and women tend to process information

A variety of marketing studies have established that women often utilize information more completely than men (Darley and Smith, 1995; Meyers-Levy and Maheswaran, 1991; Meyers-Levy and Sternthal, 1991; Showkat and Grimm, 2018; Tsichla et al., 2016). They are more sensitive to subtle cues (Meyers-Levy and Loken, 2015), to the interrelationships between pieces of information (Daltrozzo et al., 2007; Putrevu, 2001; Wirth et al., 2007), as well as to anomalies (Daltrozzo et al., 2007; Meyers-Levy and Maheswaran, 1991; Meyers-Levy and Sternthal, 1991; Wirth et al., 2007). In addition, studies show that females have better visual (Heisz et al., 2013) as well as verbal recognition (Meyers-Levy and Maheswaran, 1991) and that they engage in more extensive meaning, or semantic, elaboration (Meyers-Levy and Maheswaran, 1991; Meyers-Levy and Sternthal, 1991; Wirth et al., 2007). Other research has demonstrated that they focus more on contextual information than men (Gilligan, 1982; Kahai et al., 2023; Paraskeva et al., 2012; Stoet, 2017).

In contrast, men tend to be more selective in the way they evaluate information. As the marketing studies show, they tend to depend on the apparently most relevant or salient clues, those that point to a consistent inference, or on existing schemas (Darley and Smith, 1995; Meyers-Levy, 1986; Meyers-Levy and Maheswaran, 1991; Meyers-Levy and Sternthal, 1991; Showkat and Grimm, 2018; Tsichla et al., 2016). Consequently, they are more likely to miss subtle information and distinctions.

Men will sometimes resort to more comprehensive processing, for instance, in the presence of inconsistent information, however their threshold for inconsistency is considerably higher than for women (Meyers-Levy and Maheswaran, 1991; Meyers-Levy and Sternthal, 1991). Several experiments suggest that men do encode all the information below awareness, but some of it becomes accessible only when their usual simplification strategy is not up to the task (Meyers-Levy and Sternthal, 1991). For example, after being exposed to the test material, men were able to bring all of it to awareness under some test conditions but not others. Rather than comprehensiveness, their preferred strategy appears to favor efficient processing, which can function to amplify their potential agency.

The female strategy of using information comprehensively may extend between different modalities. An intriguing series of studies (Meyers-Levy and Zhu, 2010) indicated that women tend to bring both cognitive—or descriptive—responses and raw emotional—or hedonistic—responses, to bear on their evaluation, likely consistent with their high density of interhemispheric connections. Men, in contrast, preferred to focus only on one or the other. Specifically, those who tested high on the “need for cognition” tended to focus only on the conceptual information, while those who tested low tended to focus only on the raw emotional impact. The researchers demonstrated this with studies of music and of visual design.

Although these and many other marketing studies lend support to the view that men and women apply different cognitive strategies to product evaluation (for a review, see Meyers-Levy and Loken, 2015), only a smattering of studies have taken advantage of brain imaging technology to investigate these findings in greater detail. In a landmark brain imaging study, Wirth et al. (2007) demonstrated women’s greater sensitivity than men’s to the interrelationship between different pieces of information. The researchers used EEG, which can capture many rapid brain responses because of its ability to register changes in brain rhythms in real time (Bell and Cuevas, 2012). When shown pairs of words, some related, such as *table* and *chair*, and some unrelated, such as *coffee* and *window*, women exhibited a significant difference in EEG activity between reading related and unrelated pairs. Men, in contrast, differentiated much less between related and unrelated pairs, lending support to conclusions from behavioral studies that women tend to engage in a deeper semantic, or meaning elaboration (Meyers-Levy and Maheswaran, 1991; Meyers-Levy and Sternthal, 1991).

### Does women’s additional processing occur above or below awareness?

The different strategies that structure how men and women tend to process information are unconscious (see also Gigerenzer, 2023), guided by metacognitive signals that shape cognition (Damasio, 1994; Isenman, 2018; James, 1890; Mangan, 2001). However, the more complete information utilization and enhanced elaboration called for by the female strategy could conceivably occur either below or above awareness. Indeed, there is ever-increasing acceptance that much of cognitive processing can occur non-consciously (Hung et al., 2023; Kouider and Faivre, 2017), and some evidence suggests that women may be more proficient at certain basic aspects of it. An early study (reported in McGuinness and Pribram, 1979) found that women scored higher at extracting meaning from words presented subliminally—or below awareness. Other findings indicate that women take in more implicit environmental information than men—that is, information that they do not focus on consciously (Judge and Taylor, 2012; Kahai et al., 2023; Mosso et al., 2020).

In the EEG study by Wirth and colleagues mentioned above, the larger female difference in response to related vs. unrelated word pairs occurred in a specific EEG peak (N400). Current evidence suggests that changes in this peak may reflect processing that occurs below awareness (Hohlfeld et al., 2015; Kiefer, 2002). This conclusion is supported by a somewhat similar EEG study by Daltrozzo et al. (2007). They found a comparable gender difference in the same peak, but, in addition, they found another difference in a later peak that is generally associated with *conscious* semantic processing. However, with this later peak the gender difference was reversed with men differentiating related vs. unrelated words more than women. This finding suggests that recognizing the relationship between words in a pair tends to occur later and at a conscious (or more conscious) level for men, while for women it appears to occur sooner and at an unconscious (or less conscious) level. It lends support to the idea that women’s more comprehensive use of information than men’s may potentially depend on the processing of its inherent interrelationships below awareness.

### Slower cognitive closure can allow women to capture more potential information

Another difference in information processing between the genders, which is consistent with the view that women favor comprehensive, and men simplified and selective information processing, is that they tend to have different relationships to cognitive closure (Benko and Pelster, 2013; Kim et al., 2007; van den Bos et al., 2013; Zanini et al., 2024). This is suggested perhaps most clearly by a recent meta-analysis (Zanini et al., 2024) that confirms that women tend to perform differently than men on the Iowa Gambling Task (Bechara et al., 1994). The task was designed to model the role of unconscious cognition and emotion, and thus of intuition, in many if not most real-life decisions; it is the test most often used to assess decision-making ability clinically. Several comparable studies in rats using an analogue of the task have found similar results (van den Bos et al., 2013).

In human studies, subjects choose a card from any of 4 decks for 100 trials and win or lose play money depending on the cumulative magnitude of the rewards and penalties listed on the cards they pick (Bechara et al., 1994); they are told the goal is to accumulate money. Unbeknownst to the subjects, the decks are fixed. The two “advantaged” decks are arranged to give small rewards and small penalties, but choosing consistently from them allows players to come out ahead. On the other hand, choosing consistently from the two “disadvantaged” decks, which give double the rewards but more than double the penalties, will leave players in debt; one confers a lump sum penalty after every 10 picks from it, whereas the other allots more frequent penalties that add up to the same amount. The players are also asked every 10th card, if they noticed anything about how the decks are constructed.

On average, women begin to choose consistently from the advantaged decks 40–60 card picks *later* than men (van den Bos et al., 2013). They take longer to get a hunch that some of the decks are better, and fewer can say why this is so when the task ends after 100 card picks. This is, in part, because they continue to choose from the disadvantaged deck that gives an extremely large penalty every 10th card. If the task is extended to 200 card picks, women will begin to choose consistently from the advantaged decks but still much later than men. The large individual variation within each gender indicates that gender is only one factor determining behavior. However, the task tends to progress differently for men and women, and in addition high-scoring men score *higher* than high-scoring women, which together are suggestive of a gender-based biological influence (van den Bos et al., 2013).

The initial phase of the gambling task when subjects are probing the different decks is characterized by uncertainty and exploration. Women without awareness of doing so tend to delay closure and continue to explore, whereas men tend to come to closure more quickly and begin to exploit the pattern. The male strategy is clearly more efficient when the underlying conditions do not change—here, the underlying rules that structure the decks (van den Bos et al., 2013). Yet, importantly, the extra information women collect as they continue to monitor the different options can be critical in less stable situations. It can alert them that the underlying conditions themselves are undergoing change (van den Bos et al., 2013)—or to the existence of possible larger, more general or more nuanced patterns.

The gambling studies also highlight that the more comprehensive information utilization women tend to engage in can depend on information gathered bit-by-bit over time, since as the players explore the decks, they sample each one more or less discontinuously. In contrast, in the marketing studies by Meyers-Levy and her co-workers reported earlier, the information was presented at an explicit level and in a single batch. Moreover, because the acquisition of information about the decks happens at an implicit rather than explicit level, these findings add support to the view that much of the additional information processing women engage in can occur below awareness.

Various explanations have been put forward to explain gender differences in the gambling task. One is that men’s brains tend to focus on the long-term magnitude of gains and losses, while women’s brains focus on the frequency of gains and losses as well as the long-term payoff (Hooper et al., 2004; Reavis and Overman, 2001).^1^ Brain imaging studies detect important gender differences in the response of a crucial value-assigning region of the brain—the orbital frontal area (van den Bos et al., 2013)—which focuses men primarily on long-term payoff and women on the frequency of wins and losses in the various decks as well as the ultimate payoff. Animal studies suggest that these gender differences may depend on early life exposure to testosterone (van den Bos et al., 2013). Moreover, brain activation is largely right-lateralized for men, whereas it is largely left-lateralized for women (Bolla et al., 2004). Likewise, lesions in the right orbital frontal region tend to interfere with men’s decision-making abilities, whereas lesions in the left orbital frontal region tend to interfere with women’s decision-making abilities (Reber and Tranel, 2017; Tranel et al., 2005). Another explanation for gender differences in the gambling experiment argues that women continue to give more salience than men to the small losses that occur with picking from the advantaged decks. Imaging studies indeed show that the executive circuits in the brain have less control over emotional circuits in women as they engage in the task (Garrido-Chaves et al., 2021), at least under ordinary conditions.

Interestingly, under stress conditions focused on performance, women did better than they usually do in the gambling task, and men worse than they usually do (Preston et al., 2007; van den Bos et al., 2009). In fact, women often outperformed men under these conditions. Consistent with such observations, van den Bos et al. (2009) found that increases in the stress hormone cortisol in response to the stress correlated in female subjects with enhanced performance in the gambling task, up to a point. But for male subjects, increases in cortisol correlated with decreased performance (van den Bos et al., 2009). The female tendency to delay closure under ordinary conditions and to continue to collect information may alert them earlier to clues that the underlying conditions are changing, which can then work together with the ability to function more efficiently than usual under the resultant stress. This combination of skills may be especially important going forward in a world that seems increasingly subject to disruptive changes. However, stress generated by negative stereotypes—called stereotypic stress—has been shown to decrease female performance in the gambling task (Villanueva-Moya and Expósito, 2021). This emphasizes the importance of the workplace environment in supporting women and their potential intuitions, and perhaps especially in times of stress.

Women’s preference for more extensive information processing and their tendency to delay closure in many non-stress situations may reflect a lack of confidence (Beyer, 1998; for reviews, see Gentile et al., 2009; Lirgg, 1991). This conclusion is consistent with many studies that have shown women to be more cautious in their information processing than men (for reviews, see Charness and Gneezy, 2012; Eckel and Grossman, 2008). Yet another, not necessarily contradictory reading, as suggested by the marketing studies reported earlier, is that it reflects their underlying comprehensive focus. It also can be seen as a reflection of their caring focus. In this case, however, it would reflect *epistemic* caring, i.e., caring more about getting it just right than reaching closure–what, interestingly, Albert Einstein referred to as *conscience* (Hermanns and Einstein, 1983).

From the traditional male perspective, delaying closure and acknowledging not knowing may be interpreted as a sign of weakness, which in organizational parlance can be read as ‘incompetence’ (Anderson and Brion, 2014). Although these attitudes might be slowly changing, we are concerned that some women may deny this side of their feminine self and are reluctant to admit to not knowing even to themselves so they can feel competitive and efficient in the male-dominated world. Yet in simply acknowledging not knowing, we implicitly, if not explicitly, turn more fully toward the unconscious mind and its surprisingly complex and sometimes far-sighted intuitive potential (Beck, 2021; Isenman, 2018).

### Men may be more dependent on pre-conceptions than women

Another aspect of the difference in information processing tendencies suggested by the marketing studies is that in many situations men may be more dependent on pre-conceptions than women. Men’s tendency for selective focus on the apparently most important or salient pieces of information and on general schemas gives pre-conceptions a more central role in determining what they attend to and how they behave. Pre-conceptions are essential to meaning making, and they are almost always present (Clark, 2015). They support efficient cognition by bringing past experience to bear on current perceptions and decision making, as shown by the gambling experiments and many other studies (Clark, 2015; Gallagher, 2007).

However, pre-conceptions also have a potential downside. They can direct focus to sub-optimum or even misleading cues with respect to the current situation (Castel et al., 2007; Hammond et al., 1998) or, as the gambling experiment illustrates, away from clues that change is underway. Thus, men’s stronger dependence on pre-conceptions, which can help them to make meaning faster, also makes it somewhat easier for distortions to enter the process. With women, more complete information uptake (Meyers-Levy and Loken, 2015), heightened sensitivity to its inherent interrelationships, anomalies (Daltrozzo et al., 2007; Meyers-Levy and Loken, 2015; Wirth et al., 2007) and to context (Gilligan, 1982; Kahai et al., 2023; Paraskeva et al., 2012; Stoet, 2017), as well as willingness to delay closure (Benko and Pelster, 2013; Zanini et al., 2024; Zanini et al., 2024), can somewhat lessen the potential limiting influence of pre-conceptions and support the veracity as well as the depth of their intuitions.

## Part III: Women’s receptivity to emotional and bodily feelings can enhance their intuition

Because of women’s need for more complete information processing and its sequelae, including their focus on context (Gilligan, 1982; Kahai et al., 2023; Paraskeva et al., 2012; Stoet, 2017), they tend to immerse themselves in the current situation, which can help them to see more of what is there. Likewise, a woman’s subjective experience—which includes her emotional and bodily experience—may provide her with especially important information about the object of her attention (Offermann and Foley, 2020; Sharabiani, 2021; Vial and Cowgill, 2022). Men, in contrast, are somewhat more likely to perceive a world of separate people, objects, and events (Daltrozzo et al., 2007; Wirth et al., 2007). They also have a tendency to assume that what they perceive is independent of their subjective experience (Belenky et al., 1986; Lakoff, 2008; Polanyi, 1974). In contrast to the male stance of being an observer in a largely impersonal, objective world, the female stance is more receptive and immersive.

### The importance of bodily experience in intuition

Awareness of bodily sensations has more impact on the experience of women than men. The importance of body sensing, often called *interoception*, and images involving the body are increasingly appreciated in intuition research (for example: Bas et al., 2022; Damasio, 1994; Gendlin, 1982; Isenman, 2018; Tantia, 2011). But maximizing their usefulness in terms of surfacing and understanding one’s intuitions hinges to some extent on one’s level of bodily sensitivity (Bas et al., 2022). Experimental studies confirm that women are more focused on interoception than men (Alfano et al., 2023; Grabauskaitė et al., 2017). This enhanced attention to emotional and visceral signals in conjunction with their proposed tendency to process more below awareness (Alfano et al., 2023; Booth-Butterfield and Booth-Butterfield, 1990; Dunn et al., 2010; Grabauskaitė et al., 2017; Sinclair, 2020) would give women an advantage in many aspects of intuition.

Bodily sensation can influence intuition in three separable ways (Isenman, 2020) we will characterize below. Body experience is at the core of emotion, which causes coordinated changes in body states in response to objects in the environment significant to an organism’s survival (Damasio, 1994; LeDoux, 1995). Emotions tend to produce either approach or avoidance behavior (James, 1884), and in higher organisms can imbue the object or event with a conscious feeling of goodness or badness. Some emotion-provoking stimuli are hardwired, but many are based on learning (Damasio, 1994; LeDoux, 1995).

### Somatic markers and personal decision making

The important influence of body-based emotion and emotional learning in the cognitive realm was initially shown by another aspect of the Iowa Gambling Task (Bechara and Damasio, 2005; Damasio, 1994). The researchers found that the experience of large losses in earlier trials can function below awareness in an anticipatory way to bias future decision making. They recorded players’ galvanic skin response, or micro sweat, caused by emotional arousal, focusing on the time period before they chose cards from the disadvantaged deck. Comparison of their response to those of brain damaged patients with decision-making difficulties and specific lesions who lacked these anticipatory emotional responses (Damasio, 1994) showed that emotional arousal based on the past experience of loss activates specific higher brain centers. These centers then generate anticipatory bodily signals that help to guide behavior in an adaptive manner—the first way bodily sensations can influence intuition. The researchers called these signals, which can bias a decision either for or against an incipient choice, *somatic markers*. Somatic markers often function below awareness, although they can surface into consciousness, sometimes in the form of gut feelings (Damasio, 1994). The evidence for the influence of somatic markers on decision making, along with the other findings of the gambling task mentioned before, was critical to legitimizing the academic study of intuition.

Unpacking the complex question of how somatic markers produced by men and women lead to different behaviors in certain situations is critical to understanding aspects of intuition as it relates to gender. More research is needed to determine if there is a difference between male and female emotional arousal to actual gains or losses, to anticipated gains or losses—which serve as an approximate measure of somatic markers—and how these relate to each other during the course of the gambling task, as well as in other intuitive decision-making situations (Kandasamy et al., 2016). More sensitive measures of emotional arousal than galvanic skin response are currently available (Critchley and Garfinkel, 2017), which could be used singly and together (Garfinkel et al., 2022) to explore this as well as other questions about gender, somatic markers, and intuition.

### Somatic markers and metacognition

Somatic markers can also function as cognitive evaluative signals, or *metacognitive* signals, in other aspects of cognition than personal decision making (Damasio, 1994). These body-tinged cognitive feelings report on the validity of our own or other’s ideas, frequently below or on the fringe of awareness (James, 1890; Mangan, 2001)—the second way that bodily experience can influence intuition. They generate a subtle sense of discord or tension, or alternatively, of harmony or a reduction of tension (Isenman, 1997, 2018; Tompkins, 1962). They also act, as Damasio wrote, “covertly to highlight in the form of an attentional mechanism certain components over others, and to control in effect, the go, stop and turn signals…” that shape cognition (1994, p.190). Moreover, these metacognitive signals also likely help to guide the formation of complex intuitions by indicating below awareness that certain information does or does not fit (Damasio, 1994; Isenman, 2018). These signals also likely play a role in directing men to more efficient, and women to more comprehensive and careful, information processing.

Some of the evidence suggests that women may be more receptive to both positive and negative metacognitive signals below awareness. The brain imaging studies by Daltrozzo et al. (2007) mentioned earlier found that women tend to register an existing relationship between two words considerably sooner than men and in an earlier stage of cognition. This indicates that they may be more likely to register at least some positive metacognitive signals about connection, relationship, or fitting, prior to them surfacing into consciousness (Isenman, 2018). Consequently, these signals could have more influence on women’s forming intuitions.

Studies indicate that women are also more subject to, or perhaps more sensitive to, stop signals, consistent with the generally more cautious nature of their cognition. In studies of post-error adjustments, all subjects showed the tendency to be slower with their next response after making an error, even if they remained unaware of the error (Danielmeier and Ullsperger, 2011). The more subjects slowed down, the more likely they were to correct their errors (Fischer et al., 2016), at least those that came to awareness. Since women tend to slow down about 40% more than men after an error, they are much more likely to correct their errors than men (Fischer et al., 2016). Error correction after slowing down for errors that do not surface into consciousness has also been shown, but thus far only in typing experiments (Pinet and Nozari, 2022).

If women’s stop signals for errors that do not come to awareness also result in more correcting, this could mean that more of their imperfect, but potentially useful intuitions might be aborted before reaching consciousness. Alternatively, such stop signals could automatically redirect their forming intuitions along the lines that are more accurate without the process reaching awareness. A third possibility, which would help explain women’s greater sensitivity to subtle anomalies, is that their stop signals bring the offending element(s) along with its forming intuition to awareness (Isenman, 2018; Mangan, 2001). This would allow both the conscious and the unconscious mind to work together to resolve the contradiction or tension, often resulting in a deeper level of intuitive understanding.

### A reverse mode allows cognition to modify bodily experience

The two somatic ways of influencing intuition we have described above allow bodily sensations to enhance intuition by modifying cognition. The third way is a reverse one that allows cognition to enhance intuition by modifying bodily sensations. Here body feelings function as an expressive mode—as a language of sorts (Gendlin, 1982; Gordon, 1961; Isenman, 2020; Tantia, 2014; Wang, 2020)—and can help intuitions to surface, as well as enrich them.

Language itself tends to be embodied. When we read the word kick, for example, we produce very subtle muscular impulses in our legs (Hauk et al., 2004). Moreover, some cognitive scientists argue that physical experience is the basis for all cognitive development. According to Lakoff and Johnson (1980), cognitive development—along with language—depends on spatial and kinesthetic experiences of the body, which are transferred from the physical realm to the cognitive by metaphor. For example, think of all the metaphorical uses of the word *down* (or *up*)—such as “I am feeling *down*” and “My bank account is *down*” (Lakoff and Johnson, 1980).

Intuition can then reverse the process and use bodily sensations in place of verbal expression (Isenman, 2018). Body-based ‘language’ is often better able to convey the richness of intuitive understanding. Since it bypasses the conscious mind and its tendency to rely on conventional ways of thinking and/or on past ways of thinking, it can help—either alone or as a component of multimodal mental imagery—to impart complex novel information to awareness (Gordon, 1961; Isenman, 2018). Women, as many studies have shown, are more attuned to emotional as well as bodily, or interoceptive, feelings (Alfano et al., 2023; Grabauskaitė et al., 2017; Sinclair, 2020). Moreover, if, as we argue, women are more likely to process higher-level information below awareness than men, then they would be more likely to use this reverse, cognitive-to-body pathway.

This reverse mode can encode even abstract novel insights. Body-tinged mental images that are fanciful are particularly powerful. Since they collapse many different layers of unconscious understanding together, they can present complex multi-dimensional insights to consciousness in a deceptively simple manner (Isenman, 2018). One especially notable example is Albert Einstein’s image of riding on a light beam, which he said contained the essence of the Theory of Special Relativity (Holton, 1973). Another example is the characterization of intuition as “a blink of the eye (or I)” (Gladwell, 2005; Isenman, 1997, 2018). With its *down/up* motion pattern, the metaphor at one of its top-most levels captures the experience of intuition as a decrease, or *down*, in external vision and an increase, or *up*, in inner vision. However, the metaphor, especially in the context of the extended experience in which it initially occurred (Isenman, 1997), instead of the rapidity of thin-slicing and its resonance with the male tendency to simplify information, can be understood as stressing “not knowing” and turning toward the body and unconscious mind and their complex interactive creative potential. Consciousness can have an immediate intimation of the myriad levels of meaning encoded in such images, but it will likely decode the bounty only slowly over time (Isenman, 2018). Women may have better access to this hidden abundance, since they are more comfortable delaying closure and acknowledging not knowing than men.

### Interoception at the core of a potential intuitive channel

Given the importance of interoception to intuition, a body-based or interoceptive signaling pathway working in conjunction with emotional and cognitive pathways likely functions as an “intuitive channel.” Along with interoceptive information, this putative channel can access and integrate intuitive information about our own emotions, emotions of others, as well as intuitive information that is more cognitive in nature. As we mentioned above, women are more focused on interoceptive feelings than men (Alfano et al., 2023; Grabauskaitė et al., 2017; Sinclair, 2020) and they also may integrate more kinds of information at the same time (Meyers-Levy and Zhu, 2010; van den Bos et al., 2013). As a result, we would expect them to use the intuitive channel with its ability to integrate various types of information more than men.

A region of the brain called the insula is likely to have a major role in this channel (Bechara et al., 1994; Castelhano et al., 2019; Craig, 2011). The insula first came to widespread attention in the context of Damasio’s (1994) work on somatic markers. It consists of various highly interconnected regions that perform separable functions (Uddin et al., 2017). Its posterior region registers internal bodily sensations—or interoceptive feelings. Its more anterior regions, i.e., closer to the front of the brain, represent more “subjective” bodily sensations—for example, the perceived temperature of a probe as opposed to its actual temperature (Craig, 2011). In addition, these regions register “imagined” physical feelings; for example, the well-practiced emotional reactions and some somatic markers that no longer generate actual bodily arousal—what Damasio (1994) called “as-if feelings”—as well as more cognitive feelings, such as metacognitive feelings (Klein et al., 2013). Importantly, anterior regions also likely register the intuitive bodily signals discussed above that result from the reverse pathway. In conjunction with several other brain areas, yet another part of the insula forms what is called the *salience network* (Menon and Uddin, 2010), which determines if attention is oriented internally—toward the self—or alternatively toward cues and tasks in the external world. It also directs what specifically we attend to.

The insula with its multiple interrelated functions, as well as numerous connections with other brain areas, is active in many different aspects of sensation, emotion, and cognition (Droutman et al., 2015; Zhao et al., 2022). It is thought to play a role in the experience of sentience, and thus of self (Craig, 2011; Damasio, 1994). Some researchers argue that it is the seat of awareness (Craig, 2011; Damasio, 1994; Singer et al., 2009). This is in part because it acts as a potential meeting place where bodily sensations can shape emotion and cognition. But by the same token, it would allow emotional and cognitive influences to color interoceptive experience (Isenman, 2020; Zhao et al., 2022).

Although women have been shown to be more attuned to interoceptive experience than men (Alfano et al., 2023; Grabauskaitė et al., 2017), some studies suggest they are less accurate in registering actual interoceptive sensations (Grabauskaitė et al., 2017). Whether or not this is substantiated (Spooner et al., 2024), it leads to the interesting possibility that because of their focus on interoception women are more likely to use the reverse mode and modify their actual bodily sensations to reflect their emotional experience and cognitive information. The mixing of different kinds of information in the insula and the use of the reverse mode may account for the fact that women are more subject to psychosomatic illness than men (for a review, see Torrubia-Pérez et al., 2022). But by the same token, it would also provide women a powerful venue to interrelate bodily, emotional, and cognitive information into novel intuitions and express them with minimal intervention of the conscious mind.

A recent imaging study supports the view that there are differences in the way women and men tend to use internal bodily signals (Longarzo et al., 2021). For men, measures of attention to interoception showed a correlation with the volume of only one brain area. Called the *precuneus*, it is a multifunctional brain region that is well connected to other parts of the brain and is important in the experience of self (Cavanna and Trimble, 2006). In contrast, for women, attention to interoception correlated with the volume of multiple different brain structures involved in the construction of self, including the insula. We look forward to future studies that help to clarify the role of interoception and the insula in higher-level intuition and how it relates to gender and the construction of self.

## Part IV: Receptive intimacy, wholeness, and intuition

When researchers point to intuition’s holism, they usually refer to its non-sequential, or relational *cognitive* structure—which, as we have discussed, is indeed an integral part of holism. As noted earlier, women may use this kind of processing more than men for higher-level cognition. But the full resonance between women and intuition depends on the additional levels of wholeness that are accessible with their intuition. As we have stressed, women are receptive to other kinds of information, along with cognitive information, which they integrate all together below awareness to form a sense of the whole. Women’s intuition tends to be a product of their entire being, their emotions and their bodily sensations, as well as their cognitive capacity. However, it might surface into consciousness through any of these expressive modes or their combination (Isenman, 2018; Kündig and Sinclair, 2012).

There is also an important temporal dimension to intuition that is relevant to women and the potential multi-level wholeness of their intuition. Any experienced sense of wholeness may well be transient (Isenman, 2018). When the topic is especially important to us, our unconscious mind can continue to add connections, even if our conscious mind feels a sense of closure. As the pool of information is enlarged, the parts may undergo a reconfiguration *to form a new whole* (Adinolfi and Loia, 2022; Isenman, 2018), and this may become an ongoing process. Intuition has the tendency to build on itself, and as it does, given its relational focus—or focus on interconnections—it becomes more and more whole (Isenman, 2018). This creates the potential for an ever-growing, albeit sometimes largely tacit, expansion of knowledge.

The tendency to embed knowledge in this way may be stronger for women because their greater focus on interconnection and context (Gilligan, 1982; Kahai et al., 2023; Paraskeva et al., 2012; Stoet, 2017) makes them more open to additional information at both the conscious and unconscious level. In conjunction with their need for completeness (Meyers-Levy and Loken, 2015) and the ability to withstand the urge for closure (Benko and Pelster, 2013; Van den Bos et al., 2013), this openness can prompt them to incorporate ever more information (Fox-Keller, 1983), even if apparent conscious closure has occurred (Isenman, 2018). It sometimes may lead women to form novel, ever-more inclusive patterns at the edge of what is not yet known (Fox-Keller, 1983).

As we intimate above, the potential wholeness of intuitive knowledge is in part a consequence of the ability of the unconscious mind to register patterns that reveal themselves over time (Bechara et al., 1994; Jung, 1971). Likewise, a real relationship implies knowing the other, be it a person, object or situation, not as a static entity, but as one that continually changes. Giving careful attention to the object of interest as it changes under different conditions or evolves with time, characterizes intimacy in the sense of close familiarity (Fox-Keller, 1985). Intimacy also generally implies caring, something that may be especially true for women (Offermann and Foley, 2020). As we noted before, this caring need not be personal but could be epistemic as well. For a greater or lesser period of time, our mind and body, consciously and/or unconsciously, attend to manifold cues that signal change or alert us to additional relevant information. In management or leadership terms, this means scoping a situation holistically (Sinclair, 2010), which can sometimes allow us to foresee the ripple of interconnected consequences into the future (Hari, 2022, p. 92).

Women’s nature and nurture support receptivity and attunement to others. Because most women are biologically and culturally geared toward nurturing, they are somewhat more comfortable than men with focusing on the other (Jordan et al., 1991; Vial and Cowgill, 2022), which in part entails noticing changes under different conditions and with time. Especially primed for the helping professions (Brody and Hall, 2008; Vial and Cowgill, 2022), women at least in the past were frequently discouraged from delving into other areas. Yet, when given the opportunity, they can also extend their focus on interconnection and capacity for intimate observation, or receptive intimacy, to other types of work or activities. This has been demonstrated again and again in science, in business, and in politics.

Male intuition seems to be largely a different kind, more along the line of expert intuition. In many situations, men will use parallel interactive processing to isolate and bring to awareness the seemingly most pertinent cues, schemas or options—sometimes along the lines of thin-slicing–which can allow them to come to closure quickly (Meyers-Levy, 1986) or alternately, begin to reason logically from there. This can lead to more efficient decision making, although sometimes at the expense of ignoring information that does not fit, in the vein of Herbert Simon’s satisficing (Simon, 1956). We argue that women’s intuition can make a different, yet equally important contribution. The urge to interweave all the available information, to delay closure, register errors and information that does or does not fit, as well as to take in additional cues over time, has the potential to foster more multifaceted and far-reaching decision making, as well as a different kind of leadership.

This holism that characterizes women’s intuition is often closely associated with the right hemisphere of the brain. Although both hemispheres are involved in all activity, the right hemisphere tends to be more active than the left in complex, poorly structured, or ambiguous cognitive situations (Goel, 2014), as well as in highly context-dependent ones (McGilchrist, 2019). In a similar vein, the right hemisphere is also strongly involved in insightful and creative cognition that requires broader associations than usual (Beeman and Bowden, 2000; Mednick, 1962). In addition, it registers bodily experience more finely than the left hemisphere (Damasio, 1994) as well as many aspects of emotion (Palomero-Gallagher and Amunts, 2022) and empathy (Gupta et al., 2025), which can be especially important to female intuition. Taken together, this would suggest that women’s intuition would be especially strongly dependent on the right hemisphere. This may be true for much but maybe not all of it. The novel patterns that often characterize female intuitions, along with the lived experience (McGilchrist, 2019) and receptive intimacy that tend to underlie these patterns, likely do depend heavily on the right hemisphere. In contrast, as Goldberg (2006) claims, the recognition of already established patterns, which fuels the expert knowledge men tend to favor, may become progressively more dependent on the left hemisphere. Yet brain activation (Bolla et al., 2004) and lesion studies (Tranel et al., 2005), as we noted in the section on the Iowa Gambling task, show that much of women’s automatic decision making appears to be *left* lateralized and men’s *right* lateralized. Clearly more granular understanding is needed. In any case, what may be equally important is that the greater density of inter-hemisphere connections women tend to have (Ingalhalikar et al., 2013; Cook et al., 2023) allows them to switch more quickly between the two hemispheres than men and to integrate information more easily from both (Ingalhalikar et al., 2013; Meyers-Levy and Zhu, 2010). This ability, working in conjunction with the female tendency to grapple with complex situations below awareness, may support an even more expansive holism than we have described so far. It would allow women to integrate all their understanding *below awareness* more readily than men—encompassing intuitive as well as analytically-derived understanding (Belenky et al., 1986; Ingalhalikar et al., 2013; McGilchrist, 2019)—which may in turn generate more inclusive and complex intuitions.

## Part V: Gender, intuition, and management

In today’s turbulent economy, which is prone to disruptive change, new approaches to management and leadership are needed more than ever. As organizations face challenges brought about by rapidly evolving technology, environmental concerns, the COVID aftermath, and other yet-to-emerge crises, it is time to rethink how to make the most of human resources (The McKinsey Podcast, 2021). One of the challenges of the ongoing organizational upheaval is that traditional rational models of decision making and human resources management no longer suffice. This places an increased importance on integrating different kinds of intuition more fully into the organizational skillset (Alves et al., 2021; Hodgkinson and Sadler-Smith, 2018). As we have shown in this article, there is a natural affinity between women and the kind of intuition that is grounded in the urge for completeness and interconnection. With proper encouragement, women can unlock more of their untapped potential (The McKinsey Podcast, 2021).

Women more and more want to experience a sense of agency in the external world. Some have attempted to achieve this by acting more like men [as evidenced, for example, by the suppression of women’s intuition when they reached high echelons in STEM disciplines (McCaffrey, 2022)]. This may be especially true in leadership positions in workplaces where male culture prevails (Bierema, 2016). In the current climate of uncertainty, more than before, everyone needs a variety of different approaches and skills. For this reason, we argue, women–and the organizations that employ them–should not suppress or lose sight of their intuition, nor buy into some of the related negative stereotypes. Rather, they should tap into their special kind of intuition, which might complement the more commonly accepted ‘male kind,’ that appears geared toward efficiency and closure. Women’s intuition, which often begins with acknowledging not knowing, is a formidable asset in the quest for more far-reaching knowledge.

Encouraging women to use their intuition may also help to reshape the corporate experience. It could smooth a sometimes harsh work environment by bringing the relational qualities of interpersonal sensitivity and participative communication (Offermann and Foley, 2020) more into the foreground. Instead of shunning their intuition, women can become better acquainted with it and the deep relational urges behind it—both cognitive and personal–and begin to honor its potential agency and force. But it would require a shift of paradigm; a change would be needed both in women’s attitude toward intuition and the organization’s acceptance and support.

First of all, women would need to boost their confidence in their intuition. This can be done by becoming more attentive to the powerful in-between, or *liminal*, space from which intuitive insights tend to surface in consciousness. In other words, they can learn to focus their attention at the boundary between the conscious and unconscious mind. This supports the moment-to-moment fluctuation between knowing and not knowing, which in turn enhances the eventual, if not immediate, emergence of the unconscious material. By the same token, in accepting the experience of not knowing, a woman begins to draw more fully on all of herself. Her bodily intelligence and her conceptual intelligence start to work together more productively. This allows her to stay attuned to multiple dimensions, including the sensory, the affective, as well as the cognitive. This sense of wholeness may lead to a new level of personal authenticity, which can reinforce a more humane type of leadership with an emphasis on connectivity (Vial and Cowgill, 2022).

Yet the willingness of women to draw on their intuition at work and in public life more fully cannot occur in a vacuum. It has to go hand in hand with the willingness of the organization to legitimize and to appreciate its potential benefits. One suggestion would be to have management start encouraging all employees, not only women, to express without censoring their initial as well as intermediate thoughts about the topic under discussion. This would encourage more and more liminal thoughts and images to appear in awareness, and as they do, previously unnoticed aspects of the situation begin to emerge, greatly deepening the conversation. Such receptivity can eventually facilitate divergent perspectives coalescing into a productive novel idea or solution (Fuller et al., 2023), reflecting a collective as well as holistic approach to problem solving.

As we have stressed, the unconscious excels in integrating many factors together. For women this tends to include more intangibles such as relational and longer-term concerns that are difficult to quantify and therefore have been often excluded from traditional decisions. What women’s intuition can uniquely contribute to the organizational process is a stronger emphasis on these less quantifiable factors, without sacrificing more quantifiable ones. Corporate research has found that female board members tend to “add organizational value through the quality of their decision-making.” For example, they attend more closely to the link between pay and performance and require a clear description of roles and responsibilities. The research also indicates that “women are more likely to take a longer-term perspective and consider the organi[z]ation’s reputation, the community … and longer-term impacts” (Stirling, 2011). Including women’s views may also prove beneficial for the bottom line, as suggested by the link between corporate financial performance and the gender diversity of corporate boards (Offermann and Foley, 2020). Encouraging women’s intuition could improve the organizational climate and gradually lead to a more permanent change in organizational culture. As employees feel more included and validated, the quality of work life would be enhanced and, fueled by stronger morale, likely translate into enhanced performance.

The holistic concerns of women go hand in hand with the basic characteristics associated with their cognition. These include the tendency to process information more completely, with a special sensitivity to context, to interconnections and to anomalies, as well as the tendency to make use of body-based signals that can carry additional information potentially so important to intuition. The ability of women to immerse themselves fully in the material, to delay closure, as well as to integrate the needs of a variety of others helps to generate optimum outcomes. In this article we have found that women do have a proclivity toward an in-depth intuitive approach to situations, which if nurtured can become an important resource. Some progressive organizations have started to take seriously the expert intuition often associated with men. However, this has not yet occurred with the broader, albeit sometimes more halting, process of surfacing the often novel nonconscious knowing frequently associated with women. This leaves untapped something of great potential value to organizations as well as society. We hope this article will begin to remedy this loss.

## Data Availability

The original contributions presented in the study are included in the article/supplementary material, further inquiries can be directed to the corresponding author.

## Footnotes

^1^Differences in the structure of the task can lead to different results. Interesting animal studies by Chen et al. (2021) revealed that under specific experimental conditions, the greater female focus on frequency can result in more strategic exploration, and thus earlier exploitation of the pattern than in males, who tend to explore more chaotically under these conditions. The female focus on frequency appears critical here: male and female rats performed equally when the target cue was rewarded 100% of the time, but females performed more efficiently than males when the target cue was rewarded only 80% of the time and its alternative 20% of the time.
